# Senescent Astrocytes: A New Player in Brain Aging and Cognitive Decline

**DOI:** 10.3390/brainsci16010076

**Published:** 2026-01-06

**Authors:** Bruna Pessoa, Lívia de Sá Hayashide, Gustavo Dias, Bruno Pontes, Rafael Serafim Pinto, Luan Pereira Diniz

**Affiliations:** 1 Instituto de Ciências Biomédicas, Universidade Federal do Rio de Janeiro, Rio de Janeiro 21941-902, RJ, Brazil; 2 Centro Nacional de Biologia Estrutural e Bioimagem (CENABIO), Universidade Federal do Rio de Janeiro, Rio de Janeiro 21941-902, RJ, Brazil; 3 Instituto de Educação Médica (IDOMED), Rio de Janeiro 20071-004, RJ, Brazil

**Keywords:** astrocyte senescence, neuroinflammation, brain aging, synaptic dysfunction, neurodegeneration, mitochondrial dysfunction

## Abstract

Astrocytes are critical for maintaining brain homeostasis through metabolic support, neurotransmitter regulation, and blood–brain barrier integrity. In the aging brain and neurodegenerative conditions, astrocytes undergo functional and morphological changes that culminate in a state of cellular senescence. Astrocytic senescence—characterized by irreversible cell-cycle arrest and a pro-inflammatory senescence-associated secretory phenotype (SASP)—is emerging as a key contributor of chronic neuroinflammation and synaptic dysfunction in aging. This review examines the molecular mechanisms underlying astrocyte senescence, highlighting how persistent DNA damage responses (DDR), oxidative stress, and mitochondrial dysfunction disrupt essential astrocytic functions (e.g., glutamate uptake, K^+^ buffering, and metabolic coupling with neurons). These senescent changes in astrocytes lead to impaired synaptic plasticity and contribute to age-related cognitive decline. Collectively, astrocytic senescence represents a pivotal and targetable mechanism in age-related neurodegeneration, and therapeutic strategies aimed at eliminating senescent cells or modulating the SASP hold promise for restoring synaptic function and promoting healthy brain aging.

## 1. Introduction

Astrocytes are the most abundant glial cells in the CNS and are critical for maintaining cerebral homeostasis. Traditionally considered passive support cells, astrocytes are now recognized as active participants in numerous physiological processes essential for brain function. They provide structural and metabolic support to neurons, regulate synaptic transmission, maintain the blood–brain barrier (BBB), and play a key role in neurotransmitter recycling and ion homeostasis [1].

One of the most crucial functions of astrocytes is their involvement in energy metabolism. They uptake glucose from the bloodstream and metabolize it into lactate, which is subsequently shuttled to neurons as an energy substrate. This astrocyte–neuron metabolic coupling is vital for sustaining neuronal activity, particularly during periods of high demand. Additionally, astrocytes regulate extracellular levels of glutamate, a major excitatory neurotransmitter, by reabsorbing it from synaptic clefts and converting it to glutamine, which can be recycled back to neurons [2].

Astrocytes are also key players in maintaining the BBB, a highly selective barrier that protects the brain from harmful substances while allowing the passage of essential nutrients. By interacting with endothelial cells and pericytes, astrocytes help regulate the permeability of the BBB and ensure its integrity [3]. Moreover, they modulate the immune response in the brain by releasing cytokines and chemokines that influence the activity of microglia and peripheral immune cells [4].

Astrocytes are particularly vulnerable to the effects of aging and age-related diseases. As the brain ages, astrocytes undergo functional and morphological changes that compromise their ability to support neuronal health [5]. These changes include a decline in their metabolic efficiency, impaired glutamate clearance, and reduced antioxidant capacity, all of which contribute to a toxic environment for neurons. In aging and neurodegenerative diseases, astrocytes often become senescent, further exacerbating their dysfunction.

Importantly, astrocytic senescence should not be regarded as a uniform or homogeneous cellular state. Emerging evidence indicates substantial heterogeneity in senescent phenotypes depending on brain region, developmental stage, biological sex, species, as well as the nature, intensity, and duration of the senescence-inducing stressor [6,7]. Astrocytes display marked regional and functional diversity under physiological conditions, and this intrinsic heterogeneity is further amplified during aging and disease. Consequently, senescence-associated features such as mitochondrial dysfunction, oxidative stress burden, DNA damage signaling, and the composition of the senescence-associated secretory phenotype (SASP) may vary significantly across experimental models and pathological contexts. Accounting for this heterogeneity is necessary to ensure robust interpretation of experimental findings and to guide the translational applicability of astrocyte-targeted interventions aimed at modulating senescence.

This review explores the cellular and molecular mechanisms that drive astrocytic senescence, with a particular focus on the interplay between mitochondrial dysfunction, oxidative stress, and persistent DNA damage. Emphasis is placed on how the SASP of astrocytes disrupts neuronal homeostasis, highlighting the roles of inflammatory mediators, proteases, and growth factors in sustaining chronic neuroinflammation and promoting synaptic degeneration. Overall, astrocytic senescence emerges as an important and unifying axis linking aging, neuroinflammation, and neurodegeneration, and a deeper understanding of these processes is critical for the development of innovative strategies to promote healthy brain aging and reduce the burden of age-related neurodegenerative disorders.

## 2. Molecular Mechanisms of Cellular Senescence

Cellular senescence is primarily characterized by cell cycle arrest in G1, or possibly in G2 [8,9], but it is also accompanied by alterations in the SASP [10,11], changes in morphology, epigenetic modifications [11], functional alterations, and changes in gene expression patterns [12], among others. Thus, upon entering a senescent state due to an external or internal stressor, the cell is effectively preventing the propagation of damage to its daughter cells [13].

Moreover, although senescent cells are unable to proliferate, they remain metabolically active and resistant to signals that would normally induce apoptosis. In other words, they do not proliferate, but they actively suppress apoptotic signals [8] through the overexpression of anti-apoptotic proteins, such as BCL-W and BCL-XL [8]. Cellular senescence can be triggered by different stimuli: physiological, such as during embryogenesis, telomere shortening, development, or tissue repair [8,9]; or pathological, such as DNA damage, oncogene activation, oxidative stress, and mitochondrial dysfunction [9,11,14,15]. Therefore, cellular senescence represents an initially protective response, acting as a safeguard mechanism. However, chronic senescence can become detrimental, as the accumulation of senescent cells during aging can lead to effects such as cancer development, chronic inflammation, and immune deficiencies [9,15].

In addition to the characteristic halt in proliferation, senescent cells can also exhibit a range of morphological, molecular, and functional alterations that distinguish them from proliferating cells. Among these typical changes, increased lysosomal activity, evidenced by elevated expression of the senescence-associated β-galactosidase (SA-β-Gal) enzyme—a lysosomal enzyme encoded by the GLB1 gene, whose activity becomes detectable at pH 6 due to the increased number and size of lysosomes—is currently considered the most widely used biomarker for identifying senescent cells [9,10,15,16], as its activity rises once cells enter a senescent state. Another classic marker of senescence is the development of a SASP, characterized by profound alterations in the secretion patterns of cytokines and inflammatory factors (such as IL-1α, IL-1β, IL-6, and IL-8), as well as growth factors and tissue remodeling proteins, including Transforming Growth Factor-β (TGF-β) and Matrix metalloproteinase (MMPs) [9,13,17]. Additionally, other modifications that may be linked to the senescent cell phenotype include enlarged and flattened morphology, alterations in chromatin structure with increased chromosome condensation [9,14,16], and mitochondrial dysfunction, which may even contribute to triggering the SASP characteristic of senescent cells [15,18,19].

Different markers of cellular senescence have been observed in various cells of the Central Nervous System (CNS). In senescent astrocytes maintained in culture, for example, overexpression of senescence markers such as SA-β-gal activity and increased levels of p16INK4a, p21, and p53 is commonly observed, along with elevated levels of pro-inflammatory factors characteristic of the SASP, such as IL-6 and MMP-3, as well as the astrocyte activation marker Tissue Inhibitor of Metalloproteinases-1 (TIMP-1) [20,21,22,23]. Another characteristic finding is the reduction of lamin B1, a structural protein of the nuclear lamina essential for maintaining nuclear integrity, whose loss is considered a robust marker of astrocytic senescence; consequently, nuclear deformations are observed, evidenced by a loss of nuclear circularity [24]. Furthermore, it has been shown that senescence induction via doxorubicin, a chemotherapeutic agent that induces cell cycle arrest, in both murine and human astrocytes, also triggers activation of the DNA damage response, evidenced by the recruitment of phosphorylated H2A.X (γ-H2AX) and p53-binding protein 1 (53BP1) [23]. Additionally, in a study using senescent murine astrocytes, mitochondrial dysfunction was observed through alterations in mitochondrial morphology and respiration, as well as increased organelle fragmentation and blockage of the autophagic pathway, ultimately leading to impaired mitophagy and accumulation of damaged mitochondria [25].

To date, there is no universal marker capable of unequivocally identifying senescent astrocytes, which makes their characterization a conceptual and methodological challenge. Rather than representing a single, fixed state, astrocyte senescence is a multifactorial and dynamic phenotype whose features vary depending on the inducing stimulus, duration of exposure, tissue context, and cellular background. Consequently, the reliable identification of senescent astrocytes requires an integrated evaluation of multiple morphological, molecular, and functional features, rather than reliance on any single isolated biomarker. Accordingly, Table 1 summarizes the principal and most widely used markers associated with senescent astrocytes, providing a practical framework to support their identification and interpretation across experimental contexts.

Throughout this review, several features attributed to astrocyte senescence partially overlap with characteristics commonly associated with reactive astrocytes, making the distinction between these two phenotypes particularly challenging. Reactive astrocytes represent a heterogeneous and highly plastic response to CNS perturbations, including injury, inflammation, infection, and neurodegeneration [35]. This state is characterized by extensive transcriptional, morphological, and functional remodeling, frequently marked by upregulation of GFAP and other intermediate filaments, activation of inflammatory signaling pathways, and context-dependent alterations in astrocytic homeostatic functions [36,37]. Importantly, astrocyte reactivity is not restricted to acute pathological conditions; aging itself is associated with a progressive increase in reactive astrocyte states, as shown by large-scale transcriptomic and histological studies showing enrichment of inflammatory and reactive astrocyte signatures in the aged brain [38,39].

While reactive astrocytes typically arise as an adaptive and potentially reversible response aimed at limiting tissue damage and supporting repair, senescent astrocytes constitute a fundamentally distinct cellular state. Astrocyte senescence is defined by stable and irreversible cell-cycle arrest, persistent activation of the DNA Damage Response (DDR), sustained expression of senescence markers such as p16INK4a and p21, loss of lamin B1, and the establishment of a chronic, self-amplifying SASP [40]. Whereas astrocyte reactivity encompasses a spectrum of context-dependent states that may resolve once the triggering insult is removed, senescence is intrinsically irreversible and is associated with long-term functional decline.

A comparative overview highlighting both the key differences and areas of overlap between senescent and reactive astrocytes is provided in Table 2. Notably, despite these conceptual distinctions, it remains unclear whether astrocyte senescence represents a downstream consequence of chronic astrocyte reactivity or whether senescent astrocytes actively promote or sustain reactive phenotypes in the aging brain. Several disease-associated molecules known to induce astrocyte reactivity, including α-synuclein [41,42], tau [43,44], and Amyloid-beta (Aβ) [45,46,47], have also been shown to trigger cellular senescence, suggesting a potential mechanistic convergence between these processes. Moreover, prolonged exposure to inflammatory mediators during aging may drive a continuum in which reactive astrocytes progressively acquire senescent features, as inflammatory cytokines have been reported to induce senescence-like phenotypes in astrocytes [48]. Disentangling these relationships will require future studies with improved temporal resolution, lineage tracing approaches, and multimodal molecular profiling to clarify the causal links between astrocyte reactivity, senescence, and brain aging.

Beyond programmed senescence—which has beneficial effects, is transient, and occurs in a genetically regulated manner during physiological processes such as embryogenesis and development [49], acting, for example, as a signaling hub that orchestrates tissue growth and patterning in embryonic structures like the apical ectodermal ridge [50]—physiological forms of senescence are also observed in adult tissues, such as during wound repair. In this context, it has been shown that senescent fibroblasts and endothelial cells appear transiently in the injured tissue and promote wound closure through the secretion of Platelet-Derived Growth Factor-AA (PDGF-AA), a SASP component that induces myofibroblast differentiation to support healing [51]. Physiological senescence has also been described in processes involving cellular plasticity and reprogramming [52]. Other forms of senescence, in turn, may be subdivided according to the triggering stressor.

Replicative senescence is a type of physiological senescence that occurs due to the progressive shortening of telomeres triggered by successive cell divisions. This happens because, owing to the semiconservative mechanism of DNA replication, the ends of chromosomes cannot be fully copied. To mitigate this issue, chromosome ends are protected by telomeres—repetitive DNA sequences that are progressively lost with each cell division; in most somatic cells, telomerase activity is insufficient to compensate for this loss [8,53]. When telomeres reach a critically short length, they become recognized as double-strand breaks, leading to the recruitment of DDR activation mechanisms within the nucleus and triggering cell-cycle arrest pathways, ultimately driving the cell into senescence [11,14].

Stress-induced premature senescence is a form of premature senescence that occurs independently of telomere shortening. It is triggered by various pathological stressors that activate the DDR, resulting in permanent cell-cycle arrest. Among these stressors are DNA damage [54], oxidative stress [55], mitochondrial dysfunction [56], ionizing radiation [57], genotoxic agents [58], and oncogene activation [59].

**Figure 1:**
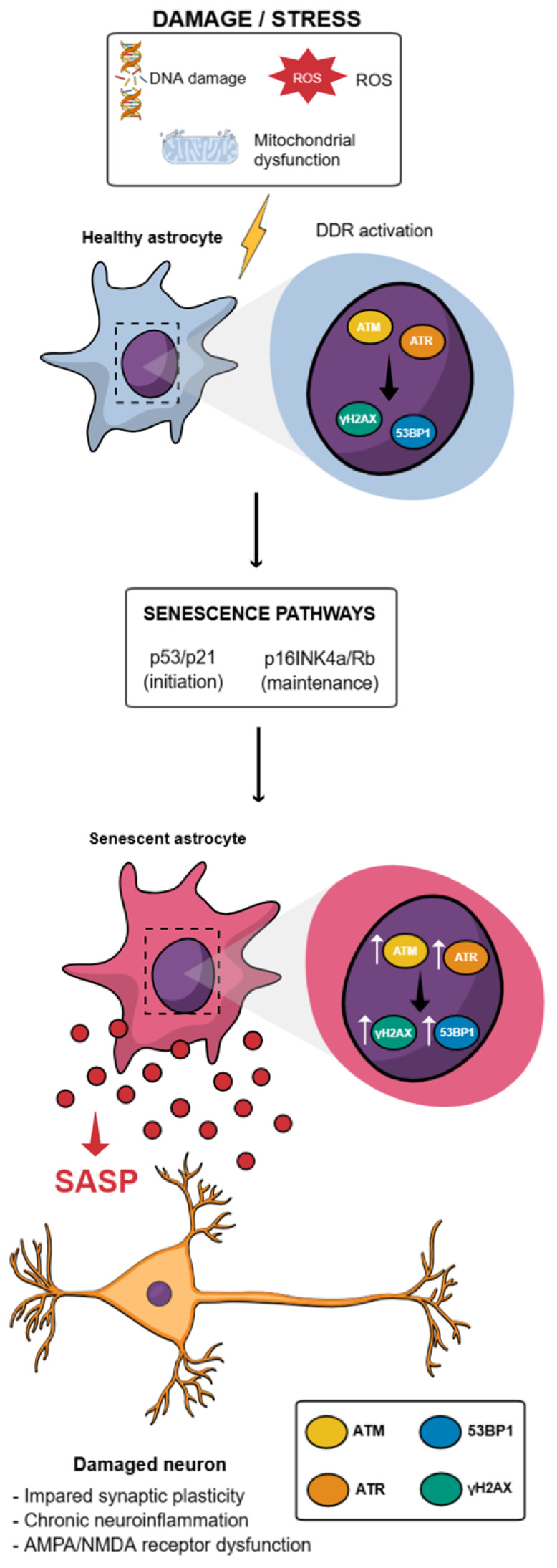
Astrocytic senescence pathways and downstream neuronal dysfunction. Schematic representation of the molecular and cellular cascade leading from stress and damage to astrocytic senescence and neuronal impairment. DNA damage, oxidative stress and mitochondrial dysfunction trigger activation of the DNA damage response in healthy astrocytes, involving key sensor kinases and signaling hubs. Persistent DDR engagement initiates and sustains senescence programs through the p53/p21 pathway, associated with senescence induction, and the p16INK4a/Rb pathway, which contributes to the maintenance of senescence. These alterations promote the secretion of a senescence-associated secretory phenotype, which acts in a paracrine manner to disrupt neuronal homeostasis, leading to impaired synaptic plasticity, chronic neuroinflammation, and dysfunction of AMPA and NMDA receptor signaling.

In any case, regardless of the trigger inducing senescence or the cell type in which it occurs, these cellular responses converge on the activation of distinct yet interconnected checkpoint pathways controlled by p53/p21 and/or p16INK4a/ Retinoblastoma protein (RB) which are associated with cell-cycle arrest and maintaining the senescent phenotype [8,60] (Figure 1).

## 3. DNA Damage Response-Driven Cell Cycle Arrest in Senescence: The p53/p21 and p16INK4a/Rb Pathways

These pathways, p53/p21 and p16INK4a/Rb, are engaged in response to activation of the DDR, in which the sensor kinases ataxia telangiectasia mutated (ATM) and ataxia telangiectasia and Rad3-related (ATR) detect genomic DNA breaks or replication-associated lesions and initiate a signaling cascade that culminates in the stabilization and activation of the transcription factor p53 [61]. Once activated, p53 binds to DNA and induces the expression of the cyclin-dependent kinase (CDK) inhibitor p21^Cip1/Waf1^, which blocks CDK activity and prevents Rb phosphorylation, thereby promoting cell-cycle arrest at the G1/S transition [15,62]. Thus, the p53/p21 pathway is more strongly associated with the initial phase of senescence, acting as an immediate response to genotoxic stress, whereas the p16INK4a/Rb pathway tends to be activated later and more stably, being essential for the maintenance and irreversibility of the senescent state [63]. Both pathways converge to maintain Rb in its hypophosphorylated state, thereby preventing the transcription of genes required for cell-cycle progression [64,65].

It is important to highlight that, unlike quiescent cells—which enter a reversible resting state (G0) in the absence of proliferative stimuli—senescent cells remain metabolically active but are permanently arrested in the cell cycle. This irreversibility stems from the sustained activation of CDK inhibitors from the KIP/CIP (p21, p27) and INK4 (p16, p15, p19) families, which maintain cell-cycle arrest even after the initial stress has been resolved.

The p53/p21 pathway therefore constitutes the primary mechanism for senescence induction in response to genotoxic stressors such as telomere shortening, DNA damage, oxidative stress, or oncogene activation [66,67]. Persistent DDR activation promotes the phosphorylation of p53 by ATM/ATR, leading to its stabilization and transcriptional activation. As the “guardian of the genome,” p53 regulates the expression of antiproliferative genes—particularly p21, encoded by *CDKN1A*—which inhibits cyclin-CDK complexes (CDK2, CDK4, CDK6) and keeps Rb in a hypophosphorylated state, thereby repressing the transcription of E2F-regulated genes [64]. This repression is essential for converting persistent damage signals into a stable proliferative arrest, marking the transition from a proliferative or quiescent state to the senescent phenotype. Unlike quiescence, where p53 and p21 activation is transient and reversible, chronic p53 activation—often sustained by persistent DDR—consolidates p21 expression and permanent cell-cycle arrest [68].

Importantly, accumulating evidence indicates that astrocytes are particularly vulnerable to persistent DNA damage and chronic activation of the DNA damage response under conditions of aging, oxidative stress, and neurodegeneration. Studies in aged human and murine cells have reported increased DNA damage markers in astrocytes, including γH2AX foci [23,27,30], 53PB1 [20,23,27,28], sustained ATM and p53 signaling [20,23], suggesting an inefficient resolution of DDR in this glial population. Given the limited proliferative capacity of adult astrocytes, prolonged DDR activation is more likely to drive a senescence-like state rather than apoptosis, characterized by stable cell cycle arrest and metabolic activity. In experimental models, genotoxic insults such as oxidative stress, irradiation, or chemotherapeutic agents have been shown to induce p53 and p21 expression in astrocytes [20,23], accompanied by reduced proliferative capacity and acquisition of senescence-associated features [33]. Moreover, persistent DDR signaling in astrocytes has been linked to the activation of pro-inflammatory pathways, including NF-κB, thereby coupling genomic instability to the development of a senescence-associated secretory phenotype. Together, these findings support the notion that DDR-driven activation of p53/p21 signaling represents a relevant and biologically meaningful mechanism contributing to astrocytic senescence and its pathological impact on the aging and diseased brain.

The p16INK4a/Rb pathway, in turn, plays an important role in the maintenance and stabilization of senescence, and has been implicated in the long-term persistence of cell-cycle arrest following the early p53/p21-mediated phase [69]. The *CDKN2A* gene, which encodes p16INK4a (as well as p14ARF through an alternative reading frame), acts as a potent tumor suppressor by directly inhibiting CDK4 and CDK6, thereby preventing the formation of cyclin D–CDK4/6 complexes associated with Rb hyperphosphorylation. As a result, Rb remains bound to E2F and blocks the transcription of genes required for G1/S transition [64]. This sustained activation of p16INK4a consolidates cell-cycle arrest independently of the p53 pathway, characterizing late senescence and contributing to the accumulation of senescent cells during aging and in degenerative diseases [70]. In the CNS, astrocytes exhibit a marked age-associated increase in p16INK4a expression, which has been linked to reduced proliferative capacity, acquisition of senescence-associated features, and functional impairment during brain aging and neurodegenerative conditions [20,24,27,29,30,32].

Although the p53/p21 and p16INK4a/Rb pathways can act independently, they often operate cooperatively and redundantly to ensure permanent cell-cycle arrest [71]. In many contexts, senescence is initiated through the p53/p21 pathway and later stabilized by sustained activation of the p16INK4a/Rb axis. This transition ensures the maintenance of the senescent phenotype even after the initial damage has been resolved or p53 is inactivated [63]. The co-expression of p21 and p16 in senescent cells suggests that both pathways may act in parallel or sequentially to reinforce cell-cycle arrest [67]. This redundancy represents a protective mechanism that prevents re-entry into the cell cycle, thereby ensuring the irreversibility of senescence and preventing malignant transformation. The continuous relevance of these pathways is highlighted by studies showing that simultaneous inactivation of p53 and Rb reverses the senescent phenotype and enables re-entry into the cell cycle [72]. Finally, the age-associated increase in p16INK4a expression further supports the chronic and stable contribution of this mechanism to maintaining the senescent phenotype [70,73].

Notably, our group was among the first to establish and characterize a robust protocol for inducing astrocytic senescence using the chemotherapeutic agent doxorubicin, demonstrating activation of the DDR, increased p21 and p53 expression in human astrocytes, and a marked reduction in proliferative capacity, thereby confirming stable cell cycle arrest. This phenotype is accompanied by a pronounced pro-inflammatory profile, with elevated expression of cytokines including MMP3, IL-6, and IL-1β, indicative of a robust SASP in human astrocytes [23]. Together, these findings establish a novel and well-characterized in vitro model of doxorubicin-induced senescence in both murine and human astrocytes, underscoring its relevance for investigating the cellular mechanisms driving age-related neuroinflammation and neurodegeneration.

## 4. Chronic NF-κB Activation Contributes to the SASP

One of the most striking aspects of cellular senescence is the establishment of the SASP, characterized by the continuous release of pro-inflammatory cytokines such as IL-6, IL-1α, IL-1β, and IL-8, as well as chemokines, growth factors, and matrix-remodeling proteases [8,12]. In astrocytes, senescence is similarly associated with a pronounced pro-inflammatory secretory profile, with increased expression and release of cytokines such as IL-6, IL-1β, TNFα, and matrix metalloproteinases including MMP-3, supporting the existence of an astrocyte-specific SASP [23,24,26,28,29,30,31,32].

Far from being a passive phenomenon, the SASP represents a highly regulated and dynamic secretory program whose composition varies according to the cell type, the inducing stimulus, and the time elapsed since senescence onset [13,74]. Temporal profiling studies have shown that its expression is controlled at multiple levels, including transcriptional and translational regulation, mRNA stability, and secretory mechanisms, and amplified by autocrine and paracrine feedback loops, which render this response robust and long-lasting [70,75]. Among the central regulators of this phenomenon, the transcription factor NF-κB stands out as the main controller of the transcription of most pro-inflammatory SASP mediators [8,17,76].

Not only linked to cell-cycle arrest, the persistence of DDR is also one of the main factors underlying the chronic activation of NF-κB during senescence [70]. In this context, damage sensors such as ATM and PARP-1 form a signaling axis that activates the IκB kinase (IKK) kinase complex, resulting in the amplification of NF-κB transcriptional activity and promoting the expression of inflammatory chemokines and cytokines that compose the SASP, such as CCL2, thereby consolidating the pro-inflammatory phenotype of senescent cells [77,78]. Multiple additional pathways converge on NF-κB in senescent cells, including p38MAPK, TGF-β–TAK1, and RIG-I/MAVS [12,79], as well as reactive oxygen species (ROS) ROS-derived stimuli that enhance IKK complex activity or directly modulate the DNA-binding capacity of p65, the transcriptionally active subunit of NF-κB, thereby amplifying the transcription of inflammatory genes [80]. Another crucial mechanism is the cyclic GMP-AMP synthase (cGAS–STING) pathway, which can activate IKK in response to the cytosolic accumulation of genomic or mitochondrial DNA, although the contribution of this pathway to NF-κB activation varies among different cellular models and remains under debate [81]. As a result of these converging mechanisms, NF-κB remains chronically activated in senescent cells, functioning as a central transcriptional hub for cytokines, chemokines and MMPs that constitute the SASP [15,17].

Senescent astrocytes exhibit chronic NF-κB activation, which is one of the main contributors of CNS neuroinflammation. This is reflected in the exacerbated expression of inflammatory mediators such as IL-6, IL-1β, TNFα, and MMP-3, a pattern described both during aging and in senescence induced by different stressors, as well as in the increased expression of p16INK4a, p21, and p53 [21,82]. Moreover, neurodegeneration models indicate that pathological markers such as Aβ and tau significantly activate NF-κB in astrocytes, intensifying the SASP and amplifying neuroinflammation [83]. In addition to chronic NF-κB activation, the mammalian target of rapamycin (mTOR) pathway is also recognized as an important mediator of senescence induction in astrocytes [26]. These two axes are described as interconnected in the establishment of senescence, suggesting that astrocytic pathology may arise from the convergence of an inflammatory pathway (NF-κB) with a metabolic pathway (mTOR) [84].

## 5. mTOR Signaling and Metabolic Reprogramming in Senescence

As previously established, senescent cells, although unable to proliferate, remain metabolically active and display metabolic demands that differ from those of proliferative cells [85]. In this context, the mTOR pathway functions as a key metabolic integrator and stands out as one of the main regulators of cellular senescence and aging. Acting as a sensor of nutrient availability, growth factors, and cellular energy status, mTOR coordinates essential anabolic processes, including cell growth and protein synthesis [86]. Thus, its prolonged activation during senescence contributes to the morphological and functional alterations characteristic of this phenotype, such as increased cell size, accumulation of mitochondrial and lysosomal mass, and the exacerbated production of SASP-associated cytokines [18,87,88].

These alterations, in turn, remodel the cell’s metabolic state, favoring the sustained activity of mTOR—particularly of its complexes mTORC1 and mTORC2. mTORC1, which plays a pivotal role in protein synthesis and degradation, amplifies the SASP by functioning as a major translational hub for inflammation: it regulates mechanisms that promote the massive translation of secreted proteins, such as IL-1α, which in turn mediates NF-κB activation and the maintenance of the pro-inflammatory profile [89,90]. Due to this strategic role, which links dysregulated metabolism to chronic inflammation, mTOR is considered a promising therapeutic target, with inhibitors such as rapamycin capable of attenuating the pro-inflammatory SASP and improving tissue health in different cellular models [91,92].

Consistent with its functions in other cell types, mTOR also regulates key metabolic and inflammatory processes in astrocytes, integrating stress signals with nutrient and growth factor availability to modulate important cellular functions [93,94]. mTORC1 activation in astrocytes alters the expression of glutamate transporters such as Excitatory Amino Acid Transporter (EAAT1 and EAAT2), impairing glutamate uptake from the synaptic cleft and consequently compromising their neuroprotective role against excitotoxicity [95,96]. Additionally, this signaling pathway affects intracellular calcium regulation, mitochondrial function, and lysosomal mass accumulation, promoting metabolic adaptations that support the secretion of SASP-associated pro-inflammatory factors [94]. Under pathological conditions such as epilepsy, brain injury, or ischemia, mTOR hyperactivation in astrocytes is associated with astrocyte activation and increased production of inflammatory mediators, including TNF-α and iNOS, directly impacting neuroinflammation and potentially neuronal survival [93,97]. Therefore, in senescent astrocytes, mTOR activation links metabolic alterations to chronic inflammatory responses, contributing not only to the maintenance of the SASP but also to synaptic and excitotoxic dysfunctions characteristic of aging and neurodegeneration [93,94].

Despite increasing evidence linking mTOR signaling to astrocytic senescence, distinguishing mTOR activation specific to senescent astrocytes from that associated with metabolic stress or reactive astrocyte states remains challenging. Senescent astrocytes commonly exhibit mitochondrial dysfunction and compensatory metabolic reprogramming, processes that can themselves drive mTOR activation [98,99,100]. Accordingly, recent findings, including work from our group, indicate a substantial mechanistic overlap between senescence, metabolic stress, and astrocyte reactivity [25]. Thus, current evidence often reflects intertwined biological states, underscoring the need for careful interpretation and explicit consideration of validated senescence models to improve mechanistic precision

Taken together, these observations position mTOR signaling as a key interface between metabolic stress and inflammatory output in senescent astrocytes. However, mTOR-driven inflammation and SASP maintenance do not occur in isolation, as they are tightly coupled to profound alterations in mitochondrial homeostasis. Given that mitochondria are important regulators of cellular metabolism, redox balance, and innate immune signaling, the next section will focus on how mitochondrial dysfunction emerges as a defining hallmark of astrocytic senescence and a key contributor of sustained neuroinflammation and neurodegenerative vulnerability.

## 6. Astrocytic Mitochondrial Dysfunction in Aging and Cellular Senescence

Mitochondrial dysfunction is an important hallmark of aging and emerges as a key modulator of astrocyte senescence and functional decline in the aging brain. Astrocytes rely heavily on mitochondrial integrity to sustain their metabolic, homeostatic, and neuroprotective roles, including glutamate uptake, ion buffering, redox balance, and metabolic support to neurons. During aging, however, astrocytic mitochondria undergo profound structural and functional alterations that compromise these essential functions and contribute to neuroinflammation and synaptic dysfunction [101,102].

**Figure 2:**
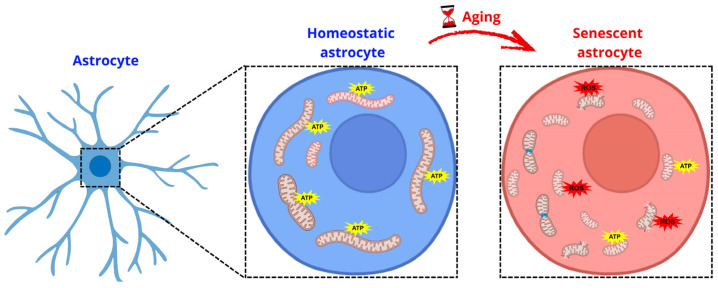
Mitochondrial remodeling and bioenergetic failure in senescent astrocytes during aging. Homeostatic astrocytes exhibit an organized mitochondrial network with preserved oxidative phosphorylation and ATP production. During aging, astrocytes acquire a senescent phenotype marked by increased mitochondrial mass driven by dysfunctional compensatory biogenesis, enhanced mitochondrial fragmentation, loss of membrane potential, reduced bioenergetic efficiency, and elevated mitochondrial ROS, ultimately leading to metabolic failure and astrocyte dysfunction.

Accumulating evidence indicates that aging astrocytes display alterations in mitochondrial morphology, bioenergetics, and quality control mechanisms (Figure 2). These changes include mitochondrial fragmentation, altered fusion–fission dynamics, impaired oxidative phosphorylation, and increased production of ROS. Studies from our group and others have demonstrated that aged and senescent astrocytes exhibit dysregulated expression of key mitochondrial dynamics proteins, including increased Dynamin-related protein 1 (DRP1) activation alongside compensatory changes in fusion mediators such as Mitofusin 1 and 2 (MFN1 and MFN2), suggesting a maladaptive attempt to maintain mitochondrial homeostasis [34]. Despite these compensatory responses, mitochondrial network integrity remains compromised, leading to inefficient energy production and increased oxidative stress.

Mitochondrial dysfunction in astrocytes is closely linked to the induction and maintenance of cellular senescence. A study suggested that mitochondria play a central role in establishing cellular senescence, particularly by sustaining the pro-inflammatory phenotype. Disruption or reduction of mitochondrial content attenuated key senescence features, including inflammatory signaling, while maintaining cellular energy through glycolysis [18]. These findings identify mitochondria as important regulators of senescence and potential targets for interventions aimed at limiting age-related tissue dysfunction.

Senescent astrocytes show reduced mitochondrial membrane potential, impaired respiratory capacity, and decreased intracellular ATP levels, reflecting a collapse in mitochondrial bioenergetic efficiency [25,30,31]. Our recent work demonstrated that senescent astrocytes exhibit a paradoxical phenotype characterized by increased mitochondrial mass and biogenesis markers concomitant with reduced mitochondrial functionality, suggesting that enhanced biogenesis may not necessarily translate into improved bioenergetic performance [25]. This mismatch between mitochondrial quantity and quality promotes the accumulation of dysfunctional organelles and exacerbates cellular stress.

Defective mitochondrial quality control mechanisms further aggravate this process. Aging and senescent astrocytes exhibit impaired mitophagy [25], resulting in the persistence of damaged mitochondria that act as chronic sources of ROS and mitochondrial danger-associated molecular patterns. These signals can activate intracellular inflammatory pathways, including NF-κB and the cGAS–STING axis, thereby reinforcing the SASP. In line with this model, our group has shown that astrocytic mitochondrial dysfunction is tightly associated with increased oxidative stress and inflammatory signaling, positioning mitochondria as important hubs linking metabolic failure to chronic neuroinflammation [103].

Etoposide-induced senescence is associated with mitochondrial dysfunction, including reduced mitochondrial membrane potential, decreased oxygen consumption, and impaired ATP production. Consistently, neurons exposed to conditioned media from etoposide-induced senescent astrocytes exhibited reduced basal respiration, ATP-linked respiration, and spare respiratory capacity, without changes in proton leak, compared to control conditioned media. Moreover, this senescent astrocyte-derived conditioned media failed to confer neuroprotection against H_2_O_2_-induced oxidative stress, resulting in increased neuronal vulnerability [31]. Corroborating this finding, a study reported that chronic exposure to senescent astrocytes impairs neuronal mitochondrial function and redox homeostasis. Using a transwell coculture model, neurons exposed to the SASP from stress-induced premature senescent astrocytes are associated with alterations in mitochondrial morphology, reduced membrane potential, disrupted GSH/GSSG balance, and compromised mitochondrial protein expression. Consistently, aged rats showed reduced oxidative phosphorylation protein levels, redox imbalance, and increased senescence markers, indicating that the senescent astrocytic microenvironment is associated with alterations in neuronal mitochondrial integrity and physiology both in vitro and in vivo [104]. Together, these findings indicate that astrocytic senescence compromises mitochondrial support and neuroprotective capacity, thereby exacerbating neuronal dysfunction.

Importantly, mitochondrial dysfunction in senescent astrocytes has direct consequences for neuronal support and synaptic homeostasis. Astrocytes with impaired mitochondrial function exhibit reduced capacity to sustain glutamate uptake and potassium buffering, as these processes are energetically demanding [29]. In line with these findings, our group reported that senescent astrocytes exhibit a reduced synaptogenic capacity and impaired modulation of neurite outgrowth, supporting the concept that astrocyte senescence may represent an early modulator of neurodegeneration during aging [24].

Conversely, these senescent astrocytes display an upregulation of glutamate transporters and glutamine synthetase [105], suggesting a compensatory or adaptive response that preserves specific homeostatic functions. These findings highlight that astrocyte senescence is not uniformly characterized by loss of function, but rather by a complex remodeling of astrocytic phenotypes, leaving open the question of whether senescent astrocytes primarily undergo functional decline or context-dependent gain of function during cellular senescence. Moreover, disruption of astrocytic mitochondrial metabolism compromises the astrocyte–neuron lactate shuttle, limiting the availability of metabolic substrates required for synaptic transmission and plasticity. Our studies and others indicate that alterations in astrocytic bioenergetics precede overt neuronal dysfunction, supporting the concept that astrocytic mitochondrial failure is an early and active contributor to age-related synaptic decline [25,34,106,107].

Collectively, these findings position astrocytic mitochondrial dysfunction as a key contributor mechanism driving senescence, neuroinflammation, and synaptic failure in the aging brain. Rather than being a passive consequence of aging, mitochondrial alterations in astrocytes actively shape the inflammatory and metabolic landscape of the CNS. Moreover, pharmacological restoration of astrocytic mitochondrial quality directly modulates cellular senescence [25]. Treatment with rapamycin promoted the clearance of damaged mitochondria, likely through enhancement of mitochondrial quality control mechanisms, and reversed key markers of astrocytic senescence. Notably, senescent astrocytes displayed increased vulnerability to mitochondrial stress induced by rotenone, indicating a reduced bioenergetic reserve and impaired stress resilience [25]. Allied to this, metformin suppresses astrocytic senescence by restoring mitochondrial homeostasis and limiting mitochondrial DNA release via Mfn2, thereby inhibiting cGAS–STING signaling. This mitochondrial–innate immune axis is pivotal for neuroprotection, as cGAS inactivation prevents astrocyte senescence, preserves dopaminergic neurons, and mitigates neurodegenerative and behavioral deficits in Parkinson’s disease models [108]. Together, these studies establish a mechanistic framework linking astrocyte senescence to mitochondrial dysfunction and subsequent deficits in synaptic regulation and plasticity.

Finally, an emerging question is whether senescent astrocytes engage alternative modes of intercellular communication to cope with mitochondrial dysfunction beyond classical paracrine signaling mediated by soluble SASP factors. Among these, tunneling nanotubes (TNTs) have gained increasing attention as actin-based conduits that enable the direct exchange of cytoplasmic content, including ions, vesicles, and organelles, between distant cells [109]. In astrocytes, TNT formation is strongly induced by cellular stressors relevant to senescence, such as oxidative stress, mitochondrial dysfunction, and persistent DNA damage responses [110,111]. Early studies reported that astrocytic TNT formation is associated to stress-activated signaling pathways, including p53 activation, and is accompanied by the transfer of mitochondria and protein aggregates [111]. Such transfer represents an intriguing yet ambivalent mechanism in the aging brain. On one hand, astrocytes can donate functional mitochondria to neurons via TNTs, enhancing neuronal survival and metabolic resilience under stress conditions [112]. This mechanism may act as short-term compensatory response, partially offsetting impaired metabolic coupling and supporting synaptic activity. On the other hand, senescent cells accumulate dysfunctional mitochondria due to defective mitophagy and altered mitochondrial dynamics [25,113]. Recent evidence further indicates that while organelle transfer through TNTs can occur between different cell types, senescent astrocytic cells preferentially act as mitochondrial donors [113]. This observation raises the possibility that TNTs may contribute to the propagation of damaged organelles or pro-inflammatory signals across astrocyte–neuron networks. Thus, while TNT formation may initially represent an adaptive response, sustained TNT-mediated transfer in the aging brain could amplify mitochondrial stress, neuroinflammation, and synaptic vulnerability. From a morphological and mechanobiological perspective, astrocytic senescence is associated with profound remodeling of the actin cytoskeleton [114]. These alterations are expected to impact on the formation, stability, and persistence of TNT protrusions. We conjecture that cytoskeletal rearrangement in senescent astrocytes may favor the persistence of TNTs, potentially shifting these protrusions from transient structures to more stable intercellular connections, thereby enabling sustained mitochondrial transfer [113]. Although this mechanistic link remains largely unexplored, emerging evidence from mechanobiology indicates that force-dependent remodeling of cell surfaces is a key determinant of protrusions [115] and may also be operative in senescent cells.

## 7. Astrocyte Senescence: Impaired Synaptic Function

Astrocytes are in close contact with pre- and postsynaptic components of neurons at so-called tripartite synapses, through which they can perform several of their multiple functions [116]. Among these functions, astrocytes not only clear neurotransmitters from the synaptic cleft—such as glutamate, via the EAAT1/EAAT2 transporters [117]—and regulate extracellular ion concentrations, but also release gliotransmitters such as D-serine [118] and provide metabolic support to neurons, for example through the lactate shuttle [119]. In this way, astrocytes directly modulate neuronal activity, influencing the strength, dynamics, and stability of synaptic transmission [120,121,122].

However, when they enter senescence, these mechanisms become profoundly compromised. Senescent astrocytes show markedly impaired neurotransmitter and ion uptake, as indicated by studies demonstrating deficits both in K^+^ homeostasis and in glutamate clearance in aged astrocytes [123]. Moreover, a preliminary study showed that the SASP of astrocytes leads to a marked reduction in the expression of the major glutamate transporters EAAT1 and EAAT2 through paracrine signaling mechanisms [124]. In parallel, senescent astrocytes display compromised metabolic support to neurons, characterized by disrupted glucose and lactate trafficking within astrocytic networks, thereby limiting the supply of energetic substrates essential for synaptic transmission and plasticity [125]. Other examples of functional decline in senescent astrocytes include reduced gliotransmitter release and impaired Ca^2+^ signaling, as the decline in Ca^2+^ dynamics decreases ATP exocytosis and weakens the ability of astrocytes to modulate synaptic transmission and plasticity [126].

As indicated, the chronic inflammation driven by the SASP of senescent astrocytes acts as a directly destructive force on synaptic plasticity. Inflammatory mediators released in the SASP, such as IL-6, TNF-α, and IL-1β, decisively interfere with hippocampal Long-Term Potentiation (LTP), disrupting the cellular mechanism underlying learning and memory [127,128]. For instance, one study showed that in animals subjected to orthopedic surgery, elevated levels of IL-1β in the hippocampus were directly proportional to memory deficits [129]. Another study showed that IL-6 overexpression in mice is associated with impairments in learning and cognitive abilities [130]. These alterations have been linked to changes in the function of key glutamatergic receptors, including AMPA and NMDA receptors, which may contribute to disrupted neuronal communication and reduced stability of synaptic networks [131]. Thus, the combination of hippocampal LTP suppression by pro-inflammatory cytokines [132] and sustained hippocampal neuroinflammation—which reduces the expression of plasticity-related genes and compromises long-term spatial memory [133]—manifests as measurable memory deficits, including working memory impairments associated with increased microglial TNF-α [134].

Beyond inflammatory effects, astroglial metabolic dysfunction critically undermines the energetic support required to maintain synaptic plasticity and processes such as learning and memory. In this context, astrocytes play an essential role in supplying lactate to neurons through the mechanism known as the astrocyte–neuron lactate shuttle hypothesis, which operates in direct response to neuronal energy demands and increased synaptic activity [135]. Studies have suggested that lactate is important for maintaining hippocampal LTP, and that inhibiting its production in astrocytes may promote direct impairments in memory consolidation [136]. Convergently, excessive lactate production by astrocytes, induced by Aβ-activated microglia, also disrupts synaptic plasticity and results in spatial memory deficits [137], reinforcing that astroglial metabolic integrity is essential for sustaining synaptic plasticity and cognitive performance.

Importantly, our work further shows that, despite an apparent increase in mitochondrial density and activation of mitochondrial biogenesis, senescent astrocytes undergo a profound metabolic reprogramming toward a glycolytic phenotype [25]. This shift is evidenced by elevated extracellular lactate levels and increased hexokinase activity. When mitochondrial respiration was challenged with antimycin to force glycolytic flux, senescent astrocytes exhibited an even greater increase in lactate production, indicating a reduced metabolic flexibility and a compensatory reliance on glycolysis. These findings suggest that enhanced mitochondrial content in senescent astrocytes does not translate into efficient oxidative metabolism, but rather reflects a maladaptive response that contributes to metabolic imbalance and impaired neuron–astrocyte coupling during aging and neurodegeneration.

Moreover, the reduction in the synthesis and release of D-serine by astrocytes compromises NMDA receptor activation, interfering with the synaptic activation required for LTP induction and worsening synaptic plasticity deficits [138,139]. Thus, chronic inflammation and metabolic failure converge to create a scenario in which the brain loses its ability to sustain the synaptic adaptations necessary for effective learning and memory function.

Our group has reported that cultures of senescent astrocytes exhibit a marked reduction in their synaptogenic capacity and in their ability to induce neuronal neurite outgrowth, indicating that astrocytic senescence compromises astrocyte–neuron signaling [24]. These findings support the idea that astrocytes are actively involved in synaptic dysfunction during brain aging, rather than being passive bystanders of neuronal decline. More recently, using a model of amyloid-β-induced toxicity, we showed that restoration of astrocytic reactivity and synaptogenic potential leads to a significant reduction in inflammatory cytokine levels, an increase in synaptic density, and a reversal of amyloid-β-induced memory impairment [107]. Together, these results highlight senescent astrocytes as key contributors to age-related synaptic failure and identify astrocyte modulation as a promising therapeutic strategy for targeting brain aging and neurodegenerative diseases.

## 8. Conclusions

In this review, we focused on how the general mechanisms of cellular senescence manifest in astrocytes and how these senescent astrocytes can undermine brain health. The molecular pathways of senescence (DNA damage response, p53/p21, p16/Rb, NF-κB, mTOR) provide multiple entry points by which age or stress can convert astrocytes from supportive partners of neurons into sources of inflammation and toxicity. A key theme is that astrocyte senescence links the well-known hallmarks of aging (genomic instability, mitochondrial dysfunction, deregulated nutrient sensing, etc.) to neural circuit dysfunction. By shedding light on astrocyte-specific changes—such as loss of glutamate uptake, impaired K^+^ buffering, decreased neurotrophin support, and secretion of neurotoxic SASP factors—this review highlights astrocyte senescence as a novel target for interventions.

Beyond mechanistic insights, increasing attention has been directed toward the pharmacological targeting of senescent astrocytes as a potential therapeutic strategy in brain aging and neurodegenerative diseases. Senolytic compounds widely used in the aging field, including quercetin, dasatinib, navitoclax, and fisetin, have been shown in preclinical models to reduce the burden of senescent cells and attenuate age-associated inflammation, with emerging evidence suggesting beneficial effects on brain function. In models of aging and neurodegeneration, clearance or functional modulation of p16-positive glial cells has been associated with reduced neuroinflammation, improved synaptic integrity, and partial recovery of cognitive performance [140,141]. However, it is important to note that most available evidence remains preclinical, and direct demonstration of selective astrocyte senolysis is still limited. Moreover, indiscriminate elimination of senescent astrocytes may carry risks, including the loss of residual homeostatic functions essential for neuronal support. In this context, senomorphic strategies aimed at suppressing deleterious components of the senescence-associated secretory phenotype, rather than ablating senescent cells, may represent a safer and more tractable approach for the CNS. Targeting pathways such as NF-κB or mTOR to dampen astrocytic SASP while preserving cell viability emerges as a particularly attractive alternative, highlighting astrocyte senescence as a nuanced but promising therapeutic target.

Moving forward, a clear focus could be placed on distinguishing senescent astrocytes from merely “reactive” astrocytes in neurodegenerative diseases, and understanding how senescence in astroglia interacts with microglial activation and neuronal pathology. Unraveling these interactions will be crucial, as it could lead to combination therapies that both reduce inflammation and boost regeneration.

In conclusion, astrocytes—long the under-appreciated support cells—now appear to play a central role in brain aging when they undergo senescence. Keeping astrocytes “young” and functional may be just as important as protecting neurons themselves in the fight against age-associated cognitive decline.

## Figures and Tables

**Table 1:** Major markers of astrocytic senescence.

Category	Marker	Characteristics	References
Classical Markers	SA-β-gal	Senescence-associated β-galactosidase activity; detectable at pH 6 due to increased lysosomal number and size	[20,23,26,27,28,29,30,31,32,24,25]
	p16INK4a	Increased expression; cell cycle regulatory protein	[20,27,29,30,32,24]
	p21	Increased expression; tumor suppressor protein involved in cell cycle arrest	[20,23,26,25,33]
	p53	Increased expression; tumor suppressor protein	[20,23]
SASP (Secreted Factors)	IL-6, IL-1β and TNF-α	Elevated pro-inflammatory cytokine	[23,26,28,29,30,31,32,24]
	MMP-3	Increased matrix metalloproteinase	[23,26,27,30,24]
	TIMP-1	Elevated astrocyte activation marker	[21]
Nuclear Structure	Lamin B1 (reduced)	Structural protein of the nuclear lamina; its loss is considered a robust marker of astrocytic senescence	[23,29,30,24,25]
	Nuclear deformities	Loss of nuclear circularity and increased nuclear area resulting from Lamin B1 reduction.	[23,30,31,32,24]
DNA Damage	γ-H2AX	Increased recruitment; indicative of DNA damage response activation	[23,27,30]
	53BP1	Increased recruitment; involved in the DNA damage response	[20,23,27,28]
Mitochondrial Dysfunction	Morphological alterations	Changes in mitochondrial morphology and respiratory function	[30,32,25,34]
	Organelle fragmentation	Increased mitochondrial fragmentation	[25,34]
	Impaired mitophagy	Blockade of the autophagic pathway leading to accumulation of damaged mitochondria	[25]

**Table 2:** Key distinctions between senescent and reactive astrocytes.

Feature	Senescent Astrocytes	Reactive Astrocytes
Trigger	Persistent genotoxic, oxidative or metabolic stress; chronic DDR	Acute injury, inflammation, infection, or neurodegeneration
Cell-cycle status	Stable and irreversible cell-cycle arrest	No stable cell-cycle arrest; context-dependent proliferative capacity
Reversibility	Irreversible	Largely reversible and context-dependent
Core markers	p16INK4a, p21, p53, loss of lamin B1, SA-β-Gal	GFAP upregulation and STAT3 activation, often accompanied by vimentin and nestin expression
DDR activation	Sustained and unresolved	Transient or limited
SASP/secretory profile	Chronic SASP with IL-6, IL-1β, TNFα, MMPs	Inflammatory or protective secretome, not a canonical SASP
NF-κB activity	Chronic and self-amplifying	Context-dependent and often transient
Functional impact	Long-term loss of homeostatic support; neurotoxicity	Can be protective or detrimental depending on stimulus
Role in aging	Accumulates with age	Observed in aging and disease; not uniquely age-driven
Therapeutic implication	Target for senolytic or senomorphic strategies	Modulation rather than elimination

## Data Availability

Not applicable.

## Abbreviations

The following abbreviations are used in this manuscript:

Aβ	Amyloid-beta
ATM	Ataxia Telangiectasia Mutated
ATR	Ataxia Telangiectasia and Rad3-related
BBB	Blood–Brain Barrier
CDK	Cyclin-dependent kinase
CNS	Central Nervous System
DDR	DNA Damage Response
DRP1	Dynamin-related protein 1
EAAT1	Excitatory Amino Acid Transporter 1 (GLAST)
EAAT2	Excitatory Amino Acid Transporter 2 (GLT-1)
IKK	IκB kinase
IL-1α	Interleukin-1 alpha
IL-1β	Interleukin-1 beta
IL-6	Interleukin-6
IL-8	Interleukin-8
LTP	Long-term potentiation
MFN1	Mitofusin 1
MFN2	Mitofusin 2
MMP	Matrix metalloproteinase
mTOR	mechanistic Target of Rapamycin
mTORC1	mechanistic Target of Rapamycin Complex 1
mTORC2	mechanistic Target of Rapamycin Complex 2
PDGF-AA	Platelet-Derived Growth Factor-AA
Rb	Retinoblastoma protein
ROS	Reactive Oxygen Species
SA-β-gal	Senescence-associated β-galactosidase
SASP	Senescence-Associated Secretory Phenotype
TGF-β	Transforming Growth Factor-β
TIMP-1	Tissue Inhibitor of Metalloproteinases-1
TNF-α	Tumor Necrosis Factor-α
53BP1	p53-binding protein 1
γ-H2AX	gamma-H2AX (phosphorylated H2A.X)
